# Prognostic value of neonatal EEG following therapeutic hypothermia in survivors of hypoxic-ischemic encephalopathy

**DOI:** 10.1016/j.clinph.2021.05.031

**Published:** 2021-09

**Authors:** Tuomas Koskela, Giles S. Kendall, Sara Memon, Magdalena Sokolska, Thalitha Mabuza, Angela Huertas-Ceballos, Subhabrata Mitra, Nicola J. Robertson, Judith Meek, Kimberley Whitehead

**Affiliations:** a Research IT Services, University College London, London WC1E 7HB, UK; b Neonatal Intensive Care Unit, Elizabeth Garrett Anderson Wing, University College London Hospitals, London WC1E 6DB, UK; c Academic Neonatology, Institute for Women’s Health, University College London, London WC1E 6HU, UK; d Department of Neuroscience, Physiology & Pharmacology, University College London, London WC1E 6BT, UK; e Department of Medical Physics and Biomedical Engineering, Elizabeth Garrett Anderson Wing, University College London Hospitals, London WC1E 6DB, UK; f Centre for Clinical Brain Sciences, University of Edinburgh, Chancellors Building, 49 Little France Crescent, Edinburgh EH16 4SB, UK

**Keywords:** Spontaneous activity transients, Active sleep, Quiet sleep, Asphyxia, Brain injury

## Abstract

**Objective:**

Early prediction of neurological deficits following neonatal hypoxic-ischemic encephalopathy (HIE) may help to target support. Neonatal animal models suggest that recovery following hypoxia-ischemia depends upon cortical bursting. To test whether this holds in human neonates, we correlated the magnitude of cortical bursting during recovery (≥postnatal day 3) with neurodevelopmental outcomes.

**Methods:**

We identified 41 surviving infants who received therapeutic hypothermia for HIE (classification at hospital discharge: 19 mild, 18 moderate, 4 severe) and had 9-channel electroencephalography (EEG) recordings as part of their routine care. We correlated burst power with Bayley-III cognitive, motor and language scores at median 24 months. To examine whether EEG offered *additional* prognostic information, we controlled for structural MRI findings.

**Results:**

Higher power of central and occipital cortical bursts predicted worse cognitive and language outcomes, and higher power of central cortical bursts predicted worse motor outcome, all independently of structural MRI findings.

**Conclusions:**

Clinical EEG after postnatal day 3 may provide additional prognostic information by indexing persistent active mechanisms that either support recovery or exacerbate brain damage, especially in infants with less severe encephalopathy.

**Significance:**

These findings could allow for the effect of clinical interventions in the neonatal period to be studied instantaneously in the future.

## Highlights

• Higher power of cortical bursts after postnatal day 3 predicted worse cognitive, language and motor outcomes.
• Association between cortical bursts and outcome was independent of structural MRI findings.
• EEG may provide additional information by indexing persistent active mechanisms that support recovery or exacerbate damage.

## 1 Introduction

Hypoxic-ischemic encephalopathy (HIE) is the most common cause of acquired neonatal brain injury (Gale et al., 2018). The majority of infants survive (Jacobs et al., 2013, Lemmon et al., 2017), but with a wide range of motor, language and cognitive outcomes spanning severe impairment to functioning above the control population mean (Azzopardi et al., 2014, Marlow et al., 2005, Natarajan et al., 2013); early prediction of neurological deficits is important to allow provision of targeted interventions. Work in neonatal rats and mice indicates that recovery following hypoxia-ischemia depends upon cortical activity, especially bursts of fast oscillations (8–30 Hz) which reflect excitatory input to pyramidal neurons (Golbs et al., 2011, Hanganu et al., 2007, Hanganu et al., 2006, Minlebaev et al., 2009, Ranasinghe et al., 2015), and that either too little or excessive neural activity can be suboptimal for sensory and cognitive development (Bitzenhofer et al., 2021, Chang and Merzenich, 2003, Tolner et al., 2012, Zhang et al., 2001). The developmental role of cortical bursting interacts with sleep cycling, which modulates burst magnitude (Adelsberger et al., 2005, Seelke and Blumberg, 2010, Seelke and Blumberg, 2008); this may mediate the association between sleep and sensorimotor plasticity in neonates (Frank, 2017, Jha et al., 2005).

In human infants, the magnitude of cortical bursts and its dependence on sleep state can be assessed using electroencephalography (EEG). At University College London Hospitals (UCLH), infants who undergo 72 h of therapeutic hypothermia for HIE are continuously monitored with 9-channel EEG for at least 3 days (Azzopardi et al., 2008). We have previously shown that this clinical service has significant patient benefit, because it identifies or rules out suspected electrographic seizures (Whitehead et al., 2016a), but it is possible that EEG could also provide prognostic information (reviewed in (Ouwehand et al., 2020)). EEG features on postnatal day 3 have been reported to predict neurodevelopmental outcome (Cainelli et al., 2018, Li et al., 2013), with greater accuracy than earlier EEG findings (Hamelin et al., 2011). However, given that abnormality of EEG is associated with extent of structural brain injury on magnetic resonance imaging (MRI) (Iyer et al., 2014, Rutherford et al., 2010), we also sought to determine whether EEG provided *additional* prognostic information to structural MRI, which is a known predictor (Lally et al., 2019, Martinez-Biarge et al., 2010, Sánchez Fernández et al., 2017).

## 2 Methods

### 2.1 Infants

This project was defined as a retrospective Service Evaluation by the UCLH Research and Development Directorate and therefore individual consent from parents was not required. All clinical data review was conducted by a UCLH-affiliated, state-registered Clinical Neurophysiologist (KW).

To identify our sample, we reviewed the records of infants born between 2012 and 2016. This was the epoch of a prior service evaluation (Whitehead et al., 2016a). Selection criteria comprised gestational age ≥36 weeks, evidence of perinatal hypoxia-ischemia, clinical presence of encephalopathy, and received 72 h of therapeutic hypothermia. Neurological evaluation had to include i) ≥9-channel EEG at postnatal day 3 or later, ii) structural brain MRI, and iii) minimum 12 months formal neurodevelopmental follow-up, including at least one assessment at ≥6 months using the Bayley Scales of Infant Development-III (Bayley-III). Exclusion criteria included ongoing seizures or anti-epileptic drug (AED) exposure (last seizure or AED dose/infusion <16 h previously). These criteria generously ensure that EEG findings will be independent of these confounds: e.g. background EEG features return within 5 h of a seizure (Arkilo et al., 2013), any effect of phenobarbital on the EEG wanes 1–6 h post-administration (van den Broek et al., 2012, Deshpande et al., 2021, Low et al., 2016, Mathieson et al., 2016, Ranasinghe et al., 2015, Sharpe et al., 2020), and for midazolam 2 h post-administration (Leuven et al., 2004). (Exposure to morphine does not affect cortical bursting when other clinical factors are taken into account (Bell et al., 1993, Hartley et al., 2018), or predict neurodevelopmental outcome in infants with HIE (Natarajan et al., 2018)). We screened 92 infants for inclusion. We excluded 12 infants who died, 10 who did not have EEG at ≥ postnatal day 3 available for review, 2 who had ongoing seizures or AED exposure, and 27 who did not have sufficient follow-up. This resulted in a total sample of 41 infants (Table 1), which exceeded the minimum sample size of at least 28 infants which was pre-determined to detect with 80% power a correlation between neonatal EEG and neurodevelopmental outcome observed by (Shellhaas et al., 2017) (R^2^ = 0.26), with alpha at 0.05.

**Table 1:** Infant demographics. UCLH: University College London Hospitals.

No. of infants	41
Inborn (vs. outborn and transferred to UCLH)	34% (14/41)
Gestational age range (weeks + days)	36 + 2–41 + 4
Sex (female:male)	21:20
Median birth weight (grams)	3280 (interquartile range: 2999–3701)
Median Apgar 1 minute^1^	2 (interquartile range: 0–3)
Median Apgar 5 minutes^1^	4 (interquartile range: 4–6)
Median Apgar 10 minutes^1^	6 (interquartile range: 4–9)
Median worst umbilical cord or first pH^1^	6.90 (interquartile range: 6.79–7.00)
Median worst umbilical cord or first base deficit^1^	−16 (interquartile range: −10 to −20)
Receipt of phenobarbital (up to 40 mg/kg total as 1st line anti-epileptic drug)	46% (19/41)
Multi-channel EEG-documented electrographic seizures	34% (14/41)
Receipt of 2nd line anti-epileptic drug(s) (after phenobarbital)^2^	12% (5/41)
Encephalopathy grade as recorded on hospital discharge summary	46% (19/41) mild, 44% (18/41) moderate, 10% (4/41) severe
Bayley-III composite motor score	41/41
Bayley-III composite cognitive score	41/41
Bayley-III composite language score	38/41 (receptive communication subscale available in one further infant (n = 39))

1 Where available.

2 The 2nd line anti-epileptic drugs used on our unit are phenytoin and midazolam.

### 2.2 EEG monitoring

Ag/AgCl recording electrodes were positioned according to the modified international 10/20 electrode placement system, and included a minimum of left and right frontal (F3,F4), central (C3,C4), temporal (T3,T4) and occipital (O1,O2), as well as midline central (Cz) coverage. The reference electrode was placed at FCz and the ground electrode was placed at the frontal hairline. Target impedance of electrodes was <5 kΩ. EEG data were recorded from 0.053 to 500 Hz with a V32 amplifier using the NicoletOne recording system (Carefusion NeuroCare, Wisconsin, USA), and digitized with a sampling rate of 250 Hz. EEG monitoring continued to a median age of 99.5 h old (range 80.5–196).

### 2.3 EEG pre-processing

In one infant, a single channel (Cz) was deleted due to poor recording quality. In all infants, artefactual EEG sections were removed by visual inspection using ‘pop_eegplot’ in EEGLAB v.14 (Swartz Center for Computational Neuroscience), and were typically defined as being >500 µV and without a physiological voltage field (Stevenson et al., 2019). This resulted in recordings of mean duration 7.7 h (minimum 0.5 h, which is sufficient to characterise prognostic neonatal EEG features (Iyer et al., 2015)).

### 2.4 EEG analysis: Cortical burst power

**Fig. 1:**
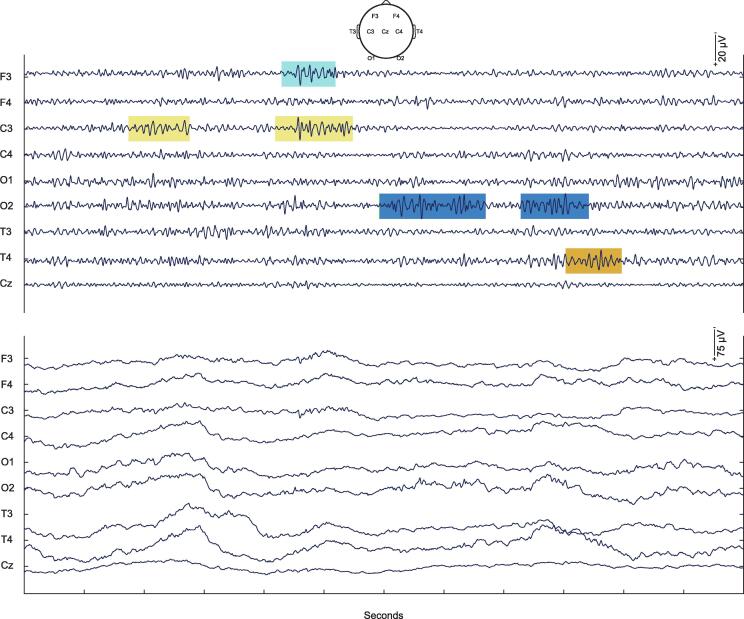
Illustrative cortical bursts in infant #1. Upper panel: Examples of automatically detected bursts of fast oscillations (8–30 Hz). Lower panel: Same section of EEG but unfiltered except for a 50 Hz notch filter.

**Fig. 2:**
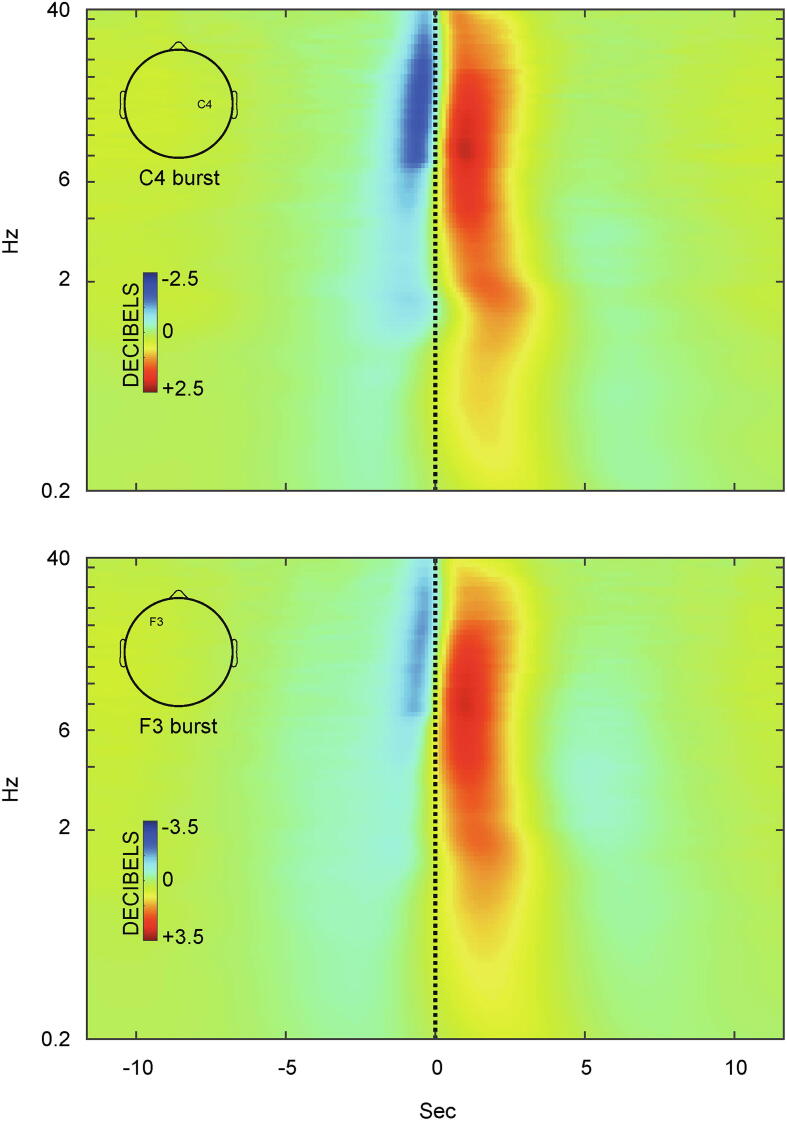
Grand average time-frequency changes associated with cortical bursts. Examples are provided for bursts identified at channels overlying different brain hemispheres and regions (upper panel: right central, C4; lower panel: left frontal, F3). Power changes between 0.2 and 40 Hz (logarithmic scale) are shown in decibels, relative to the mean power throughout the 11 s preceding burst onset (black vertical line), where increased power is shaded red and decreased power is shaded blue. Note the small ‘hotspot’ of particularly increased power at circa 8 Hz (dark crimson).

Bursts of fast oscillations (8–30 Hz) are present in the human neonatal EEG (Whitehead et al., 2016b). To identify these bursts, we calculated root-mean-square (RMS) amplitude values between 8 and 30 Hz for each EEG channel, using sliding 400-ms intervals (Antony et al., 2018, Hartley et al., 2012, Ranasinghe et al., 2015). We then extracted segments for each channel that were consecutively above a set threshold (1.5 times the standard deviation of its RMS signal over the whole recording (Antony et al., 2018)) for ≥0.5 s (Omidvarnia et al., 2014), using ‘detectevent’ in EEGLAB. For each EEG, automatically detected bursts were spot-checked to confirm validity by a Clinical Neurophysiologist (KW) (e.g. Fig. 1). To characterise the time-frequency characteristics of detected bursts (Fig. 2), we convoluted the EEG signal with a Morlet wavelet between 0.2 and 40 Hz using an increasing range of cycles (3–135), employing ‘newtimef’ in EEGLAB. For bursts detected at each channel, e.g. right frontal (F4), we extracted the power between 8 and 30 Hz at that channel over the course of the burst, and then normalised this value by dividing by its duration in seconds, using custom-written Matlab code (Koskela et al., 2021).

### 2.5 EEG analysis: Periodicity of cortical burst power

Earlier emergence of sleep cycling (e.g. postnatal day 0 vs. day 2) is associated with better neurodevelopmental outcome, but by the time point analysed here, the EEG commonly tracks the sleep cycle (Osredkar et al., 2005, Sewell et al., 2018, Watanabe et al., 1980). To investigate whether the parameters of the dependence of the magnitude of cortical bursts on sleep state predicted neurodevelopmental outcome, we used Fourier transform to visually identify the highest amplitude spectral peak of burst power across the recording, which was consistent with a sleep cycle (period <3.5 h; at a parasagittal channel for consistency with prior studies (Osredkar et al., 2005, Shellhaas et al., 2017)), using custom-written Matlab code (Koskela et al., 2021). For recordings below 4.5 h, the identified spectral peak was unreliable, because it was predicted almost completely by recording duration (r = 0.988, p < .001, Pearson correlation). Therefore, we characterised sleep cycle length using the 31 recordings of over 4.5 h duration, for which the identified spectral peak was reliable because its period and amplitude were independent of both recording duration and the amount of artefactual EEG removed during pre-processing (p ≥ 0.150).

### 2.6 Magnetic resonance imaging (MRI)

Structural MRI was performed on a 3T system (Philips Medical Systems, Best, The Netherlands) between postnatal day 4 and 13 (please see Supplementary Information for further details). MRI findings were classified according to the National Institute of Child Health and Human Development Neonatal Research Network 6-point injury score (Shankaran et al., 2017, Shankaran et al., 2012).

### 2.7 Neurodevelopmental outcome

As part of their routine clinical follow-up, infants were invited for neurodevelopmental assessments using the Bayley Scales of Infant Development, Third Edition (Bayley-III), typically at 3, 6, 12 and 24 months (Bayley, 2010). Its cognitive scale assesses abilities such as sensorimotor exploration, its motor scale consists of fine (e.g. grasping) and gross (e.g. locomotion) motor subscales, and its language scale consists of receptive (e.g. comprehension) and expressive (e.g. babbling) communication subscales. The scales are age-standardised with the composite cognitive, motor and language scales having a reported mean of 100.

The median age of the last Bayley-III assessment was 24 months (39/41 infants ≥12 months; maximum 36 months). Where the last Bayley-III assessment occurred at 6 months (2 infants), later (non-Bayley-III) standardised assessments ≥12 months confirmed the earlier Bayley-III findings: Gross Motor Function Classification System level 5 (four limb cerebral palsy) and severe global developmental delay in 1 infant with Bayley-III scores ≤ 3rd centile; normal neurological examination (optimality score 78/78) in 1 infant with Bayley-III scores within the normal range.

For a single infant who could not be assessed on the expressive communication subscale because of severe delay, for the purpose of this analysis we assigned a score of less than −3 SD from the reported mean, in line with previous literature (Anderson et al., 2010), resulting in a subscale score of 0. In two infants who could only be assessed at 24 months using one of the motor subscales (score 14 in both cases), due to lack of cooperation, for the purpose of this analysis we assigned 14 for the other subscale also, given that their 12 months Bayley-III assessments had yielded identical scores for each motor subscale.

### 2.8 Statistical analysis

To test for association of continuous variables we used Pearson and partial Pearson correlations. Group-level correlations were defined as statistically significant if p < .05, and bootstrap 95% confidence intervals did not cross zero. To examine whether continuous variables predicted a binary measure (Bayley-III composite score of <85), we conducted a receiver operating characteristic (ROC) analysis and i) calculated the area under the curve (AUC) which is a combined measure of sensitivity and specificity: an AUC of 0.5 indicates prediction no better than chance, while 1.0 would reflect a perfect predictor, and then ii) examined its coordinates to identify cut-off points which yielded optimum sensitivity with the least trade-off in specificity. Statistical analysis was performed using IBM SPSS v. 26 and custom-written Matlab code (Koskela et al., 2021).

## 3 Results

### 3.1 Higher cortical burst power predicted worse cognitive, motor and language outcomes

Across infants, cortical bursts had median duration 1.5–1.7 s for frontal, central and temporal bursts, and 2.2 s for occipital bursts, and median occurrence rate of one burst per 10–13 seconds per channel.

Cortical bursts comprised a broadband increase in power, but maximal between 8 and 30 Hz as expected, immediately preceded by a brief decrease in power between approximately 8–30 Hz just prior to burst onset (Fig. 2). For bursts identified at each channel (e.g. C4 or F3), the largest changes in 8–30 Hz power were at that channel as anticipated, although bursts involved other channels also (Supplementary Fig. 1).

**Fig. 3:**
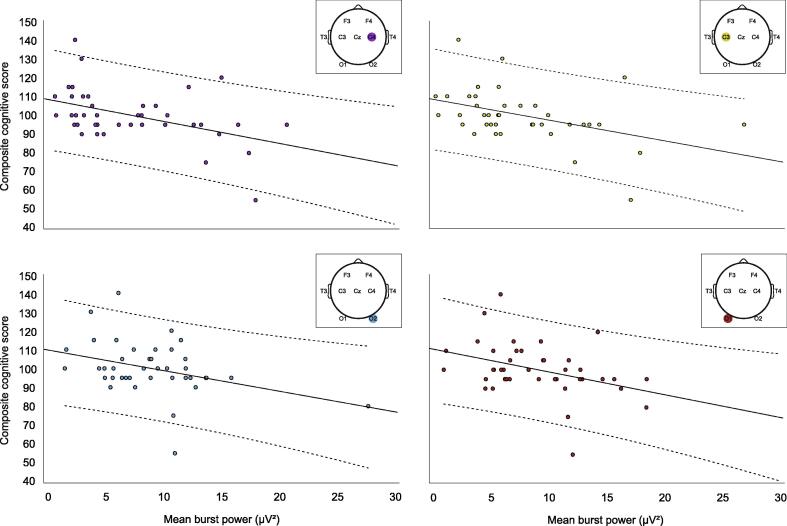
Higher cortical burst power predicted worse cognitive outcome. Lines of best fit and 95% confidence intervals (dashed lines) are plotted for associations between burst power and cognitive outcome. For conciseness, associations are plotted for the four channels with the strongest correlation coefficients (see text for details).

For bursts detected at each channel, we extracted the power between 8 and 30 Hz at that channel over the course of the burst, normalised by burst duration. Higher bilateral central and occipital and right temporal burst power predicted worse cognitive outcome (C3: r = −0.432 [−0.687 −0.129], p = .005, C4: r = −0.454 [−0.676 −0.143], p = .003; O1: r = −0.375 [−0.564 −0.178], p = .016; O2: r = −0.355 [−0.540 −0.081], p = .023; T4: r = −0.328 [−0.569 −0.119], p = .036; Fig. 3). After controlling for severity of structural MRI findings (Supplementary Information), this association remained significant for bilateral central and left occipital regions (adjusted: C3: r = −0.410 [−0.642 −0.099], p = .009, C4: r = −0.467 [−0.693 −0.120], p = .003; O1: r = −0.317 [−0.548 −0.055], p = .046), and dropped to borderline p values for right temporal and occipital regions although confidence intervals still did not cross zero (adjusted: T4: r = −0.311 [−0.564 −0.021], p = .050; O2: r = −0.303 [−0.527 −0.046], p = .057).

**Fig. 4:**
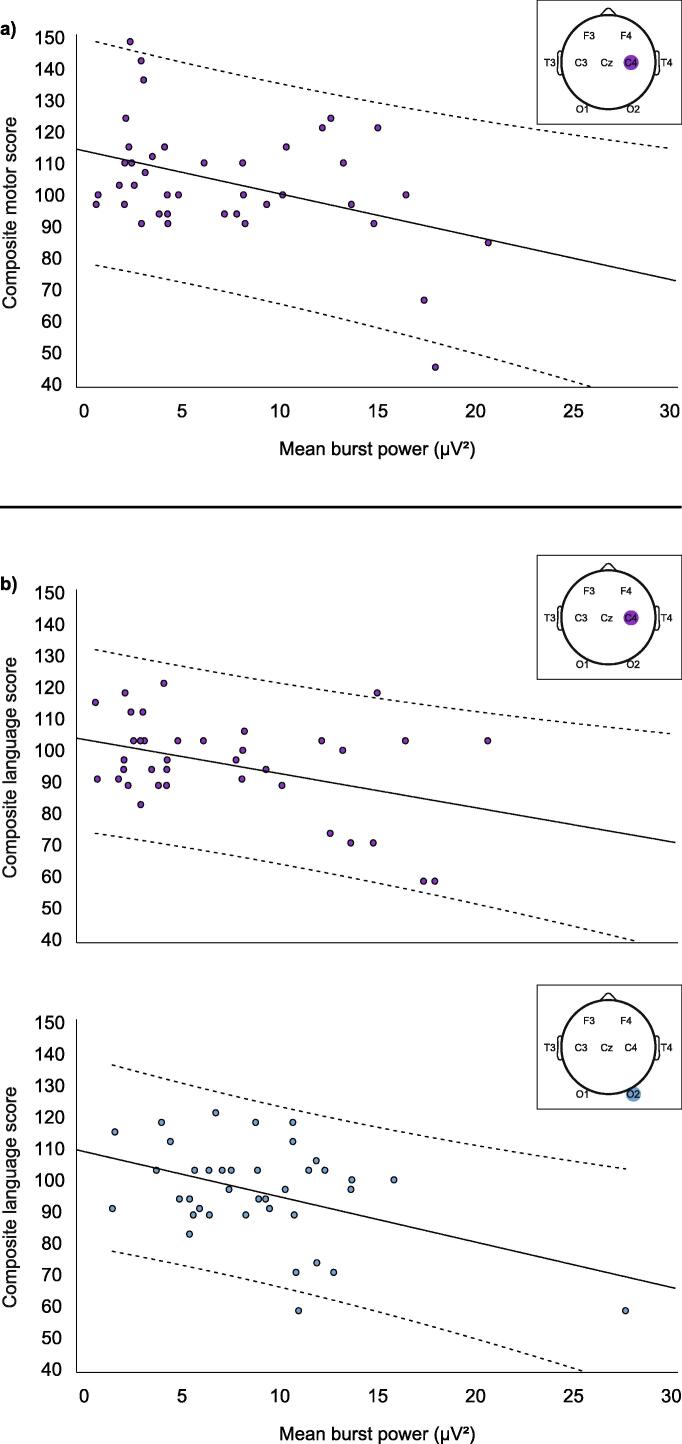
Higher cortical burst power predicted worse motor and language outcome. Lines of best fit and 95% confidence intervals (dashed lines) are plotted for associations between burst power and a) motor and b) language outcome.

Higher right central burst power predicted worse composite and gross motor outcomes (composite: C4: r = −0.409 [−0.625 −0.055], p = .009, Fig. 4a; gross: C4: r = −0.393 [−0.631 −0.032], p = .012), and this association remained significant after controlling for severity of structural MRI findings (adjusted composite: C4: r = −0.414 [−0.635 −0.057], p = .009; adjusted gross: C4: r = −0.390 [−0.621 −0.035], p = .014).

Higher right central and occipital burst power predicted worse composite and expressive language outcomes (composite: C4: r = −0.408 [−0.708 −0.026], p = .012; O2: r = −0.436 [−0.689 −0.046], p = .006, Fig. 4b; expressive: C4: r = −0.417 [−0.668 −0.097], p = .010; O2: r = −0.490 [−0.751 −0.004], p = .002). These associations remained significant after controlling for structural MRI findings (adjusted composite: C4: r = 0.403 [−0.684 −0.002], p = .015; O2: r = −0.404 [−0.657 −0.011], p = .013; adjusted expressive: C4: r = −0.411 [−0.658 −0.066], p = .013; O2 remained p < .05 but confidence interval now crossed zero: r = −0.472 [−0.723 0.026], p = .003).

Taken together, these results indicated that burst power explained unique variance in cognitive, motor and language outcomes. We also assessed the ability of burst power to predict outcome as a *binary* measure (abnormal: yes/no). The AUC for abnormal cognitive outcome was 0.95, 0.94, 0.84, 0.84, and 0.84 for the right and left central, right and left occipital, and right temporal channels respectively; using a cut-off threshold between 10.9 µV^2^ and 14.6 µV^2^, sensitivity was 100% and specificity varied between 76% and 89% for central and occipital channels, and was 66% for the temporal channel. The AUC for abnormal motor outcome was 0.97 for the right central channel; using a cut-off threshold of 17.0 µV^2^, sensitivity and specificity were 100% and 97.4%. The AUC for abnormal language outcome was 0.83 and 0.76 for the right central and occipital channels; using a cut-off threshold of 12.5 µV^2^ and 10.9 µV^2^ respectively, sensitivity and specificity were 83% and 88% for the central channel, and 83% and 79% for the occipital channel.

### 3.2 Cortical burst power was periodic

**Fig. 5:**
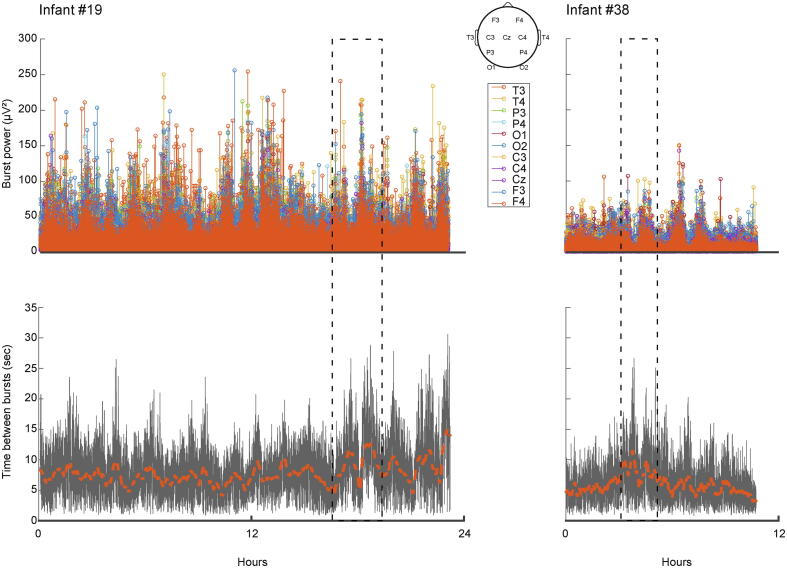
Burst power and occurrence were periodic. Upper panel: time series of burst power for infant #19 (left) with poorer outcome (composite scores 59–80 across domains) and #38 (right) with better outcome (composite scores 91–106 across domains). Lower panel: time series of time since last burst at a representative channel (C4) for the same infants. Note the positive correlation between burst power and time since last burst (good examples within dashed boxes; in these two infants correlation coefficients were r = 0.23 and r = 0.08 respectively (mean of coefficients for each channel)).

Burst power was periodic in every infant, with a mean 1.7 h cycle (range: 0.7–3.3 h). Burst power cycled in phase with the time since last burst at that channel: when the time since last burst was longer, burst power was higher (Fig. 5; mean correlation coefficient across infants r = 0.21 [range across infants 0.06 0.44] (mean of coefficients for each channel)). These data indicate the presence of sleep cycling: periods of higher burst power and longer inter-burst intervals are consistent with the tracé alternant pattern of non-rapid eye movement sleep, and periods of lower burst power and shorter inter-burst intervals are consistent with rapid eye movement sleep (Supplementary Information and Supplementary Fig. 2) (Osredkar et al., 2005, Shellhaas et al., 2017, Whitehead et al., 2018a).

Neither sleep cycle length nor the strength of the association between time since last burst and burst power predicted neurodevelopmental outcome (p ≥ 0.605 and p ≥ 0.302 respectively).

## 4 Discussion

Here we show that EEG acquired as part of routine clinical care provides additional prognostic information to an index of lesion load (structural MRI) in survivors of HIE. Cortical burst power may offer unique information by indexing persistent active mechanisms that either support recovery or exacerbate brain damage (Fleiss and Gressens, 2012, Hassell et al., 2015, Roberts et al., 2014), especially in infants with less severe encephalopathy. Cortical bursts reflect excitatory input to pyramidal neurons in neonatal animal models (Hanganu et al., 2007, Hanganu et al., 2006, Minlebaev et al., 2009). Constrained, patterned cortical bursting subserves healthy sensorimotor maturation (Molnár et al., 2020), and could explain the association between modest burst power and good neurodevelopmental outcome observed here. On the other hand, *excessive* cortical bursting may have independently mediated adverse neurodevelopment by ‘swamping’ highly plastic circuits with disorganised excitatory input (Chang and Merzenich, 2003, Zhang et al., 2001). In line with this, experimental augmentation of fast cortical activity in neonatal mice is sufficient to impair their later cognitive development (Bitzenhofer et al., 2021).

Bursting specifically overlying somatomotor cortex predicted motor outcome. In healthy infants, bursts of fast oscillations in this region are carefully choreographed and often occur somatotopically, time-locked to tactile input or somatosensory feedback from orofacial and body movements (Fabrizi et al., 2016, Whitehead et al., 2018b). As discussed above, *excessive* cortical bursting may then disrupt the orchestration of this developmental process.

Bursting overlying somatomotor and visual cortices predicted cognitive and language outcomes. Both of these cortices exhibit altered plasticity following hypoxia-ischemia in neonatal rats (Failor et al., 2010, Quairiaux et al., 2010, Ranasinghe et al., 2015). It is likely that successful integration of somatomotor, visual and other sensory information will determine higher-order abilities like cognition and language (Cainelli et al., 2018, Dall’Orso et al., 2021, Dijkerman and de Haan, 2007, Filippetti et al., 2013, Keysers et al., 2010, Nevalainen et al., 2017), and cortical activity has been implicated in the facilitation of such long-range functional connectivity (De León Reyes et al., 2019, Gibson et al., 2014, Leroy-Terquem et al., 2017, Tataranno et al., 2018).

The predictive value of EEG observed here was modest when compared to that reported for MR spectroscopy, which has recently become established as a routine clinical prognostic tool (our MR spectroscopy methodology was being optimised over the study period examined) (British Association of Perinatal Medicine, 2020, Lally et al., 2019, Mitra et al., 2019, Ouwehand et al., 2020). However, EEG can track how a prognostic biomarker such as cortical bursting is *modulated*, by factors like sleep cycling. Such temporal information may allow optimisation of the environment in future, for example to support sleep states which promote healthier patterns of cortical activity (Georgoulas et al., 2021, van den Hoogen et al., 2017). Whilst gross sleep features, like cycle length, were not associated with neurodevelopmental outcome here, detailed sleep characteristics - which can only be assessed using synchronised video and/or additional electrodes (Supplementary Fig. 2) - have been shown to predict outcome (Shellhaas et al., 2021, Shellhaas et al., 2017).

This study had some limitations. Its retrospective design restricted data availability. In addition, although 12 months outcomes - the minimum follow-up time here - closely predict later outcomes in sensorimotor domains (Hamelin et al., 2011), longer follow-up periods are advantageous for other domains such as language (Azzopardi et al., 2014, Marlow et al., 2005, Natarajan et al., 2013). Furthermore, it would have been preferable for all infants to have been followed to exactly the same age. Finally, by excluding infants who died or had ongoing seizures, the sample largely comprised infants with less severe encephalopathy. One consequence of this is that we may have been unable to detect the negative consequences of too *little* cortical bursting: persistence of almost entirely suppressed EEG activity is associated with the worst cases of HIE, when mortality and seizure burden are highest (Hellström-Westas et al., 1995, Lemmon et al., 2017, Watanabe et al., 1980). While a linear model fit the association between cortical burst magnitude and outcome here, inclusion of more severely affected infants might indicate that a non-linear, e.g. quadratic, model is more appropriate.

In summary, clinical cot side EEG recordings can provide prognostic information in real time, likely by indexing the reorganisation of cortical circuits after injury. These preliminary data suggest that in the future EEG could allow for the effect of clinical interventions in the neonatal period to be studied instantaneously.

## Declaration of Competing Interest

The authors declare that they have no known competing financial interests or personal relationships that could have appeared to influence the work reported in this paper.

## Appendix A Supplementary material

Supplementary data 1

Supplementary data 2 Grand average time-frequency changes associated with cortical bursts. Examples are provided for bursts identified at channels overlying different brain hemispheres and regions (upper panel: right central, C4; lower panel: left frontal, F3; bold black outlines). Power changes between 0.2 and 40 Hz (logarithmic scale) are shown in decibels, relative to the mean power throughout the 11 seconds preceding burst onset (black vertical line), where increased power is shaded red and decreased power is shaded blue. Note the small ‘hotspot’ of particularly increased power at circa 8Hz (dark crimson) at the channel where the burst was identified.

Supplementary data 3 Cortical burst power declined with rapid eye movements. Lower panel: Data from infant #10, showing an independent component with a scalp topography that reflected horizontal eye movements. The red-blue-red etc. repeating pattern during two periods of the stacked EEG (marked by pale blue boxes), indicated recurrent reversals of the component’s polarity as the eyes moved. Middle panel: Periods of eye movements could be identified by higher amplitude of this component (pale blue shading); zooming in on the channels close to the eyes (F7,F8) at this time showed inverted deflections consistent with horizontal saccades. Upper panel: Eye movements were associated with periods of lower burst power (shown at a representative channel (C4) on a logarithmic scale), in line with lower burst power being characteristic of rapid eye movement sleep.
