# Supplementary material for: Prognostic value of neonatal EEG following therapeutic hypothermia in survivors of hypoxic-ischemic encephalopathy
Source: Clin Neurophysiol. 2021 Sep;132(9):2091–100. doi: 10.1016/j.clinph.2021.05.031 (PMC8407358; doi:10.1016/j.clinph.2021.05.031)
Supplement: Supplementary data 1 [file mmc1.docx]

**Prognostic value of neonatal EEG following therapeutic hypothermia in survivors of hypoxic-ischemic encephalopathy**

Tuomas Koskela^a^, Giles S Kendall^bc^, Sara Memon^d^, Magdalena Sokolska^e^, Thalitha Mabuza^b^, Angela Huertas-Ceballos^b^, Subhabrata Mitra^bc^, Nicola J Robertson^bcf^, Judith Meek^b^, Kimberley Whitehead^bd^

^a^ Research IT Services, University College London, London, WC1E 7HB, UK

^b^ Neonatal Intensive Care Unit, Elizabeth Garrett Anderson Wing, University College London Hospitals, London, WC1E 6DB, UK

^c^ Academic Neonatology, Institute for Women’s Health, University College London, London, WC1E 6HU, UK

^d^ Department of Neuroscience, Physiology & Pharmacology, University College London, London, WC1E 6BT, UK

^e^ Department of Medical Physics and Biomedical Engineering, Elizabeth Garrett Anderson Wing, University College London Hospitals, London, WC1E 6DB, UK

^f^ Centre for Clinical Brain Sciences, University of Edinburgh, Chancellors Building, 49 Little France Crescent, Edinburgh EH16 4SB, UK

**Supplementary Information**

Magnetic Resonance Imaging (MRI)

The structural MRI scanning protocol included T1-weighted imaging (inversion-prepared 3D gradient echo read-out: inversion time (TI)=1465ms, repetition time (TR)=17ms, echo time (TE)=4.6ms, sagittal slice thickness=1mm, in-plane resolution=0.82×0.97mm) and T2-weighted imaging (coronal and axial, turbo spin echo: echo train length=11, TR=10721ms, TE=130ms, slice thickness=3mm, in-plane resolution=0.50×0.52mm), DTI (32 directions, b=750, echo planar imaging read-out: TR=7500ms, TE=49ms, slice thickness=2mm, in-plane resolution=2.0×2.04mm).

MRI findings were classified according to the National Institute of Child Health and Human Development Neonatal Research Network 6-point injury score (Shankaran et al., 2017, 2012).

Findings were classified as normal in 29 infants (score 0), cerebral lesions only in 8 infants (1A in 6, 1B in 2), and involving the basal ganglia and thalamus, anterior or posterior limbs of the internal capsule, or watershed infarction in 4 infants (2A in 2, 2B in 2). (No infant was assigned the worst score of 3). Severity of MRI findings was negatively correlated with cognitive outcome (r = -.445 [-.699 -.121], p = .004), composite and gross motor outcomes (composite: r = -.435 [-.694 -.062], p = .004; gross: r = -.363 [-.621 -.010], p = .021), and receptive communication outcome (r = -.376 [-.625 -.035], p = .018). Severity of MRI findings was not significantly correlated with other outcome scales, although r values were negative in all instances.

Case study: Cortical burst power declined with rapid eye movements

When pronounced horizontal eye movements occur during rapid eye movement sleep, EEG channels close to the eyes (F7, F8) can record a retinal voltage field which tracks the eye movements (Whitehead et al., 2019), which may be retrieved using independent component analysis. Two infants had enough channels (17) and data (over one hour) to decompose their EEG into its independent components (Onton et al., 2006). In one of these infants, one of the components accounting for the most variance (within the top 15 components) reflected pronounced horizontal eye movements. Eye movements were associated with periods of lower burst power, in line with lower burst power being characteristic of rapid eye movement sleep (Supplementary Figure 2) (André et al., 2010).

**References**

André M, Lamblin M-D, d’Allest AM, Curzi-Dascalova L, Moussalli-Salefranque F, Nguyen The Tich S, et al. Electroencephalography in premature and full-term infants. Developmental features and glossary. Neurophysiologie Clinique/Clinical Neurophysiology 2010;40:59–124. https://doi.org/10.1016/j.neucli.2010.02.002.

Onton J, Westerfield M, Townsend J, Makeig S. Imaging human EEG dynamics using independent component analysis. Neuroscience & Biobehavioral Reviews 2006;30:808–22. https://doi.org/10.1016/j.neubiorev.2006.06.007.

Shankaran S, Barnes PD, Hintz SR, Laptook AR, Zaterka-Baxter KM, McDonald SA, et al. Brain injury following trial of hypothermia for neonatal hypoxic–ischaemic encephalopathy. Archives of Disease in Childhood - Fetal and Neonatal Edition 2012;97:F398–404. https://doi.org/10.1136/archdischild-2011-301524.

Shankaran S, Laptook AR, McDonald SA, Hintz SR, Barnes PD, Das A, et al. Acute Perinatal Sentinel Events, Neonatal Brain Injury Pattern, and Outcome of Infants Undergoing a Trial of Hypothermia for Neonatal Hypoxic-Ischemic Encephalopathy. The Journal of Pediatrics 2017;180:275-278.e2. https://doi.org/10.1016/j.jpeds.2016.09.026.

Whitehead K, Slobodina M, Meek J, Fabrizi L. Fronto-central slow cortical activity is attenuated during phasic events in rapid eye movement sleep at full-term birth. Early Human Development 2019;136:45–8. https://doi.org/10.1016/j.earlhumdev.2019.07.007.
