# Supplementary figures and images for: Prognostic value of neonatal EEG following therapeutic hypothermia in survivors of hypoxic-ischemic encephalopathy
Source: Clin Neurophysiol. 2021 Sep;132(9):2091–100. doi: 10.1016/j.clinph.2021.05.031 (PMC8407358; doi:10.1016/j.clinph.2021.05.031)

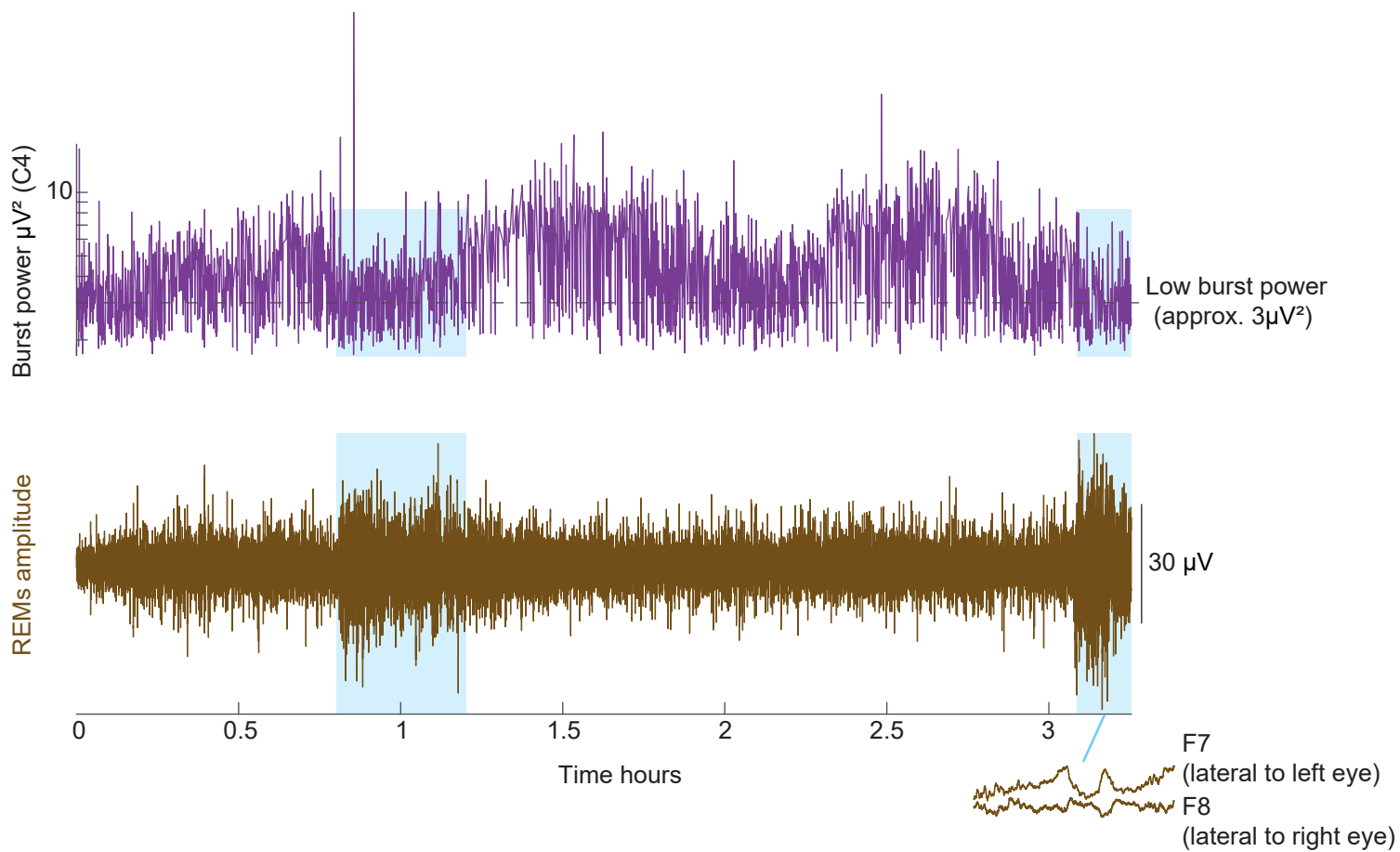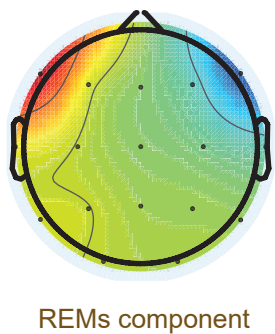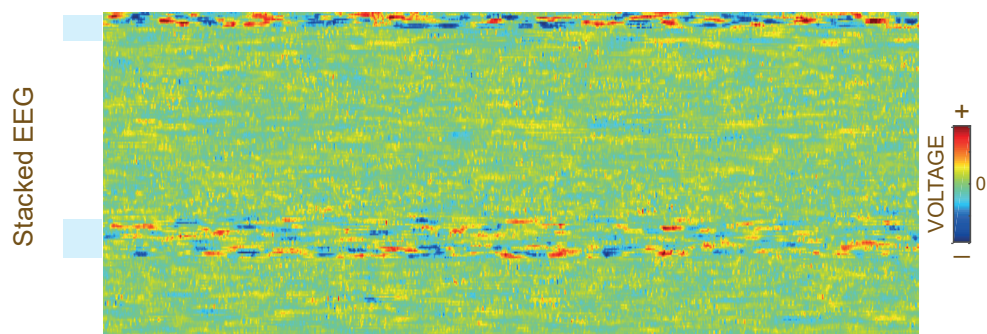

Supplement: Supplementary data 3 — Cortical burst power declined with rapid eye movements. Lower panel: Data from infant #10, showing an independent component with a scalp topography that reflected horizontal eye movements. The red-blue-red etc. repeating pattern during two periods of the stacked EEG (marked by pale blue boxes), indicated recurrent reversals of the component’s polarity as the eyes moved. Middle panel: Periods of eye movements could be identified by higher amplitude of this component (pale blue shading); zooming in on the channels close to the eyes (F7,F8) at this time showed inverted deflections consistent with horizontal saccades. Upper panel: Eye movements were associated with periods of lower burst power (shown at a representative channel (C4) on a logarithmic scale), in line with lower burst power being characteristic of rapid eye movement sleep. [file mmc3.pdf]
